# Analysing omics data sets with weighted nodes networks (WNNets)

**DOI:** 10.1038/s41598-021-93699-3

**Published:** 2021-07-14

**Authors:** Gabriele Tosadori, Dario Di Silvestre, Fausto Spoto, Pierluigi Mauri, Carlo Laudanna, Giovanni Scardoni

**Affiliations:** 1 Center for BioMedical Computing (CBMC), University of Verona, Strada le Grazie 8, 37134 Verona, Italy; 2 Section of General Pathology, Department of Medicine, University of Verona, 37134 Verona, Italy; 3 Department of Computer Science, University of Verona, Strada le Grazie 15, 37134 Verona, Italy; 4 Institute for Biomedical Technologies, National Research Council (ITB-CNR), via F.lli Cervi 93, Segrate, 20090 Milan, Italy

**Keywords:** Computational models, Data integration, Network topology, Predictive medicine

## Abstract

Current trends in biomedical research indicate data integration as a fundamental step towards precision medicine. In this context, network models allow representing and analysing complex biological processes. However, although effective in unveiling network properties, these models fail in considering the individual, biochemical variations occurring at molecular level. As a consequence, the analysis of these models partially loses its predictive power. To overcome these limitations, Weighted Nodes Networks (WNNets) were developed. WNNets allow to easily and effectively weigh nodes using experimental information from multiple conditions. In this study, the characteristics of WNNets were described and a proteomics data set was modelled and analysed. Results suggested that degree, an established centrality index, may offer a novel perspective about the functional role of nodes in WNNets. Indeed, degree allowed retrieving significant differences between experimental conditions, highlighting relevant proteins, and provided a novel interpretation for degree itself, opening new perspectives in experimental data modelling and analysis. Overall, WNNets may be used to model any high-throughput experimental data set requiring weighted nodes. Finally, improving the power of the analysis by using centralities such as betweenness may provide further biological insights and unveil novel, interesting characteristics of WNNets.

## Introduction

Systems biology is a fast developing discipline that aims at creating a unifying conceptual framework to investigate the emergent properties of complex biological systems^1,2^. An important application of systems biology falls in the field of biomedical sciences^3^. Here, a very important direction of investigation is offered by the growing availability of proteomics data sets^4,5^ coupled with increasingly reliable protein-protein interactions (PPI) networks^6,7^. These massive data sets allow constructing very large PPI networks that, in turn, are fundamental to understand how complex sets of PPI influence cells phenotype and behaviour^8,9^. However, the construction of treatment-specific or individual networks including experimental information is still a challenge^10–12^. Many modelling approaches have been proposed to achieve this goal and can be divided into two main categories. On the one side, there are network models focusing on weighting edges^13,14^. These approaches clearly lack a way of modelling experimental information about nodes since the focus is on the interactions between nodes, i.e. on the edges. On the other side, there are network models aiming at weighting nodes. Previous work by Dopazo et al.^15^, Tang et al.^16^, and Li et al.^17^, proposed interesting node-weighting methods. Dopazo et al. designed a framework to retrieve cancer-related genes in tumour versus control networks. They used different centrality indexes to weigh and then rank nodes. Tang et al. designed a new way of predicting essential proteins based on a novel centrality, i.e. weighted degree, using protein protein interactions and gene expression data. Finally, Li et al. designed a framework, comprising novel ad-hoc centralities, for constructing and analysing nodes and edges weighted PPI networks based on a set of primary protein structure predictors and edges confidence scores. The main drawback in these approaches concerns the fact that, to rank nodes in the weighted networks, novel centrality indexes were defined. Also, none of the proposed approaches allowed the integration of experimental data into the network structure, at node level.

To overcome these limitations, we developed a novel PPI networks modelling procedure that accurately incorporates experimental information using a simple, yet powerful, approach. The first step was the definition of the theoretical framework that allowed the construction of weighted nodes networks (WNNets). Then, the new approach was validated by using an experimental high-throughput proteomics data set^18,19^ comprising 24 samples. The data set included two groups of healthy control samples (NH and NU) and two groups of treated samples (H and U). A set of proteins, shared across all samples, was initially identified and used to construct the master, not weighted, PPI network. Then, by adding new nodes, i.e. copies, and edges to the master network, 24 WNNets were constructed, and degree was computed to rank weighted nodes and investigate their properties^20^. Finally, to support the validity of experimental data weighted WNNets, a comparison with a set of randomly weighted WNNets was performed. In addition, further WNNets mathematical properties were reported (see Supplementary materials, Appendix A and Appendix B).

**Figure 1:**
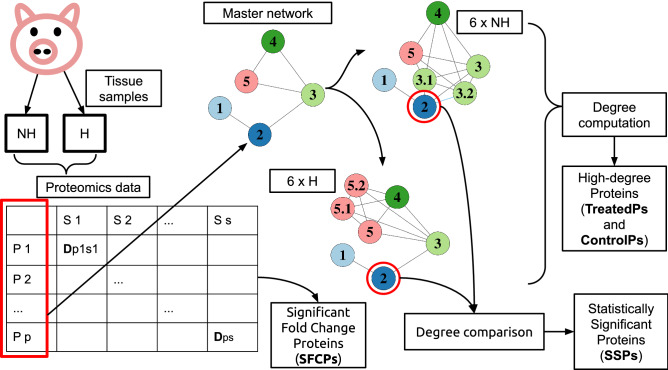
Analysis workflow. The analysis took advantage of a data set derived from myocardial tissue samples. Two types of samples were obtained: healthy control samples (NH or NU), and samples that suffered a heart attack and underwent a specific treatment (H or U). 24 samples were analysed using mass spectrometry to obtain a proteomics data set, represented as a matrix with a column for each sample and a row for each protein. The experimental information were used to construct WNNets and to compute fold change. Fold change was used to determine statistically significant differences between groups (SFCPs). The list of proteins, highlighted by the red box on the left, was used to construct the master network. The master network allowed the construction of 24 WNNets, each representing a tissue sample. Finally, degree was computed and high-degree proteins (TreatedPs and ControlPs) were identified. Degree was also used to test differences between groups at protein level, e.g. P2 from 6 H samples was compared to the degree of its corresponding healthy control samples, i.e. P2 from 6 NH samples. This comparison, performed for each protein in the master network, provided a set of treatment-specific, statistically significant, proteins (SSPs).

## Methods

### Biological data

Myocardial infarction semi-quantitative data^18^ were generated by means of Multidimensional Protein Identification (MudPIT), that is based on the combination of liquid chromatography and tandem mass-spectrometry (LC/LC-MS/MS)^21^.

The data set included 24 protein lists obtained by analysing samples of myocardial tissue derived from the left ventricle of farm pigs (*Sus scrofa*). Specifically, U samples included six protein lists obtained from myocardial tissues that suffered a heart attack treated with Phosphate Buffered Saline (PBS), and six protein lists obtained from healthy myocardial tissues extracted from the same animals (NU). Similarly, H samples included six protein lists obtained from myocardial tissues that suffered a heart attack, treated with FMhMSCs preconditioned with a mixed ester of hyaluronic, butyric and retinoic acids, and 6 protein lists obtained from healthy myocardial tissues extracted from the same animals (NH). It is important to note that each treated group (H or U) was coupled and compared with its corresponding healthy control group (NH or NU). The study received specific ethical approval from the Italian Ministry of Health, Department of Veterinary Public Health, Food Safety and Health Protection (Ordinance 113/2009 B and 117/2012 B)^19^.

The data that were used to construct WNNets refer to Spectral Count (SpC) which is defined as the total number of spectra, or peptides, that were identified for a given protein. SpC is widely used for proteome quantification using label-free approaches^22^. The full data set^18,19^ comprised SpC values for 1560 proteins. Each SpC was normalised using the molecular weight of the corresponding protein to let proteins being comparable within a sample. Then, the data set was filtered to remove all those proteins having an experimental value equal to zero, in at least one sample. Finally, the normalised SpCs of the 113 shared proteins were used as weights to construct the WNNets, without any further normalisation or transformation. The original data set included two groups of samples (HF and F) that were not considered for this analysis.

**Figure 2:**
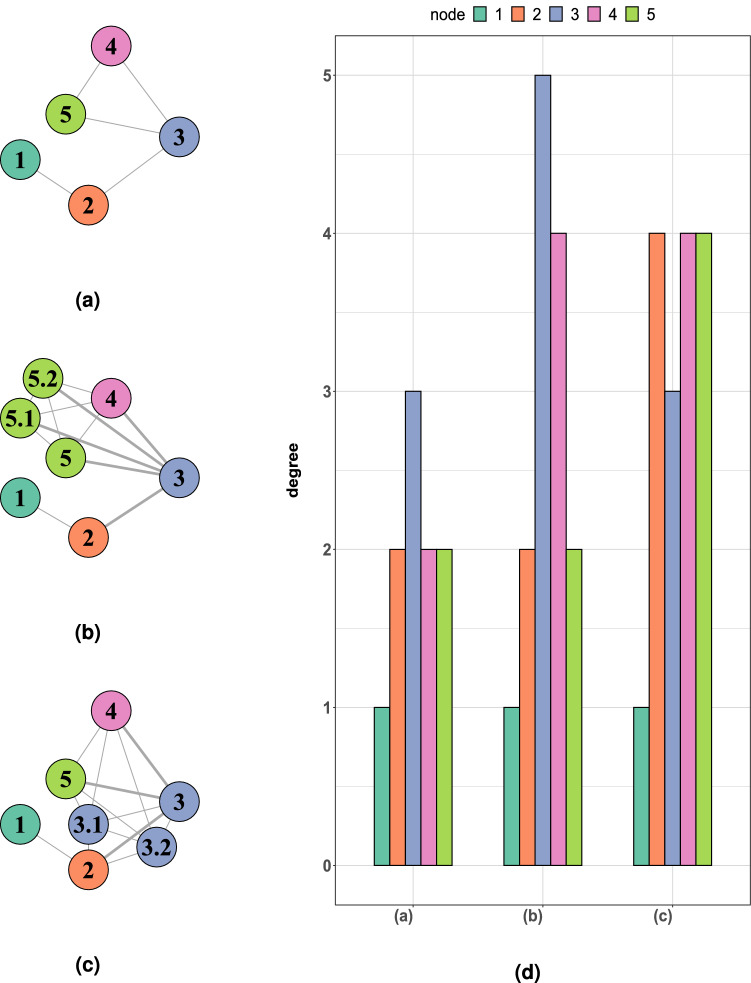
Same network, different WNNets. Once the master network was constructed **(a)**, two copies of master node 5, i.e. two new, green copies, were added. Then, these copies were connected with each other and with their master node to obtain a WNNet **(b)**. The same procedure was performed to model master node 3 and obtain a different WNNet **(c)**. Then, all the networks were analysed using degree. The bar plots **(d)** show the degree each master node scored in the master network and each of the two WNNets. The colour of each bar reflects the colour of the corresponding master node, and its copies, in the corresponding network. The length of the bar represents the master node degree.

### Network construction and analysis

Protein–protein interactions were retrieved from STRING v11, experimentally validated interactions with a score over 400, i.e. the medium confidence threshold that is set as default by STRING. The 113 shared proteins were mapped using the human interactome as a reference since *Sus scrofa* is commonly used as an animal model for several human pathologies^23,24^ and due to the higher amount of reliable interactions described for *Homo sapiens*. The resulting STRING network included 83 proteins and 157 edges, divided into three isolated components, i.e. non communicating subnetworks, comprising 79, 2, and 2 nodes, respectively. For this analysis, the biggest connected component, including 79 nodes and 155 edges, was extracted and used as master network to build the WNNets (see Supplementary materials, Appendix E).

Degree was used to compute the relevance of a master node, i.e. each node in the master network, in each WNNet. Degree, according to its original definition, measures the number of neighbours a node has^25^. In the case of WNNets, degree still measures the number of neighbours, but this number depends on the weighting that was used to construct the WNNet. Indeed, $Degree(v) = \sum _{u=1}^{m} w_{u}$ such that $w_{u} > 0$. In other words, the degree of master node *v* is equal to the sum of the weights $w_u$ of its *m* neighbours with $1 \le u \le m$. So, if master node $v_{1}$ is connected to master node $v_{2}$ whose weight is $w=5$, then, in the corresponding WNNet, $Degree(v_{1})=5$. As mentioned, degree was used to rank the master nodes. To achieve this goal, four quantiles were computed, one for each group of samples, i.e. NH, NU, H, and U. Then, those proteins above the third quantile, i.e. 75%, in their corresponding group, were considered as high-degree proteins (ControlPs and TreatedPs).

Statistically significant proteins (SSPs) were found by computing a t-test statistics with multiple tests correction. The correction was performed using the False Discovery Rate (FDR). A protein was considered SSP if its treated samples degrees (H or U) were significantly different, i.e. adjusted *p-value* $< 0.1$, if compared to its corresponding healthy controls degrees (NH or NU).

Proteins with a significant fold change increase (SFCPs) were determined by thresholding the $log_{2}( fold\ change )$ and by computing a t-test with multiple tests correction. To compute the fold change, the normalised SpCs of each protein belonging to a group, i.e. NH, NU, H, and U, were averaged and the ratio, i.e. the actual fold change, was computed as $foldChange=\frac{treated}{healthy\_control}$, for the two comparisons, NH-H and NU-U. Normalised SpCs of each protein were used to compute the adjusted *p-values*, for both comparisons. Finally, all the proteins with a fold change above or below the threshold, which was set equal to $\pm \ 1$, and with FDR < 0.1 were considered as SFCPs.

Differences between NH-H and NU-U were computed by subtracting the healthy control average degree (NH or NU) from its corresponding treated average degree (H or U), for each protein. The differences were computed as absolute values and, finally, averaged to obtain a global difference for each comparison (Fig. 4).

Upset plots were obtained by intersecting all the sets of interesting proteins, for all the comparisons, i.e. NH-H, and NU-U. Each set had a variable number of proteins depending on its cardinality (Fig. 5).

All the analysis were performed using R version 4.0.5 (2021-03-31), x86_64-pc-linux-gnu, Xubuntu 20.04. The following libraries were used: igraph^26^ version 1.2.6, RColorBrewer^27^ version 1.1-2, ggrepel^28^ version 0.9.1, UpSetR^29^ version 1.4.0, reshape2^30^ version 1.4.4, gtools^31^ 3.8.2, extrafont^32^ 0.17, and ggplot2^33^ version 3.3.3.

### Comparison to random-weighted networks

A comparison between biologically weighted WNNets and randomly weighted WNNets was performed. This analysis was designed to test whether the properties of WNNets were dependent on the master network structure, i.e. the number of its master nodes and edges, or on the weights that were used to construct the WNNets.

To achieve this goal, 100 random data sets were generated. Each random data set, comprising 24 columns, one for each sample, and 79 rows, one for each protein, was used to construct a set of WNNets. These data sets were generated by picking random values in a specific range defined as $[ minimum\ experimental - maximum\ experimental ]$. The minimum and maximum experimental values were calculated using the original weighting data set. So, for each trial, 24 WNNets were constructed using the original master network and then compared using the WNNets framework, to test the existence of SSPs. Results showed that adjusted *p-values* were negligible for all the proteins, in all the trials (see Supplementary materials, Appendix D).

### Definition of WNNets

A generic data set describing experimental data is modelled as a matrix *D* with *p* rows and *s* columns. Each $D_{i, j} \in {\mathrm{I\!N}}^{+}$ with $1 \le i \le p$ and $1 \le j \le s$ represents the experimental value for protein i in sample j. Each sample j yields a WNNet that is based on a master network.

A master network is defined as a graph $G = (V, E)$, whose nodes $V = \{v_{1}, \ldots , v_{p}\}$, called master nodes, are the *p* proteins and whose undirected edges *E* connect proteins with each other. The experimental value $D_{ij}$ is the weight $w(v_i) > 0$ of a master node $v_i$ (Fig. 1). Moreover, it is assumed that $w(v_i)$ is an integral value, for each $v_i\in V$. This assumption will be discussed later.

A WNNet is a graph $G' = (V', E')$ obtained by weighting the master nodes in *G*. Notably, *G* can be seen as a WNNet whose master nodes weights are equal to 1.

Now, consider a master node $v_i\in V$ such that $w(v_i)>1$ and define $G' = (V', E')$ such that it has $a = w(v_i) - 1$ more new nodes than *G*, that is

$$\begin{aligned} V' = V \cup \{v_1',\ldots ,v_a'\}, \end{aligned}$$

where $\{v_1',\ldots ,v_a'\}$ are nodes not in *V*, i.e. they are the copies of the master node $v_{i}$.

The edges of *G* are modified in $G'$ so that $Clique =\{v_1',\ldots ,v_a',v_i\}$ become a fully connected clique in $G'$, with new edges $I = \{(x,y)\mid x,y\in Clique \}$.

Finally, all nodes in $Clique$, i.e. the master node and its copies, are connected to the same master nodes, and their copies, that $v_i$ was connected to, namely $C = \{(x,u) \mid (v_i,u) \in E\text { and }x\in Clique \}$.

By defining

$$\begin{aligned} E'= E \cup I \cup C, \end{aligned}$$

a graph $G'=(V',E')$ is constructed. This process is repeated for each master node whose weight, i.e. its experimental value, is greater than 1. Finally, once all master nodes are processed, the result is a WNNet.

The assumption that weights are integral values is not limitative, since any graph whose master nodes have positive real weights can be translated into a graph whose master nodes have weights belonging to the natural numbers set. The use of natural numbers, instead of positive real numbers, for the weights of the master nodes, does not affect the computation of degree either, since the latter depends on the sum of the weights of the neighbours of a master node. Therefore, the addition of 1 or of a positive real number, possibly very small, contributes to the importance of a master node, which is directly dependent on the real, experimental data (see Supplementary materials, Appendix B).

As a practical example, a master network was constructed (Fig. 2a). At this point, suppose that all the experimental weights were 1, except for master node 5 whose weight was 3. To construct the corresponding WNNet, two copies of master node 5, i.e. copies 5.1 and 5.2, and edges connecting the copies to the master node, were added to the master network to model the actual weight of master node 5, i.e. 3 copies (Fig. 2b). Edges were also added, connecting the two new copies 5.1 and 5.2 to the master nodes neighbours.

The same procedure was applied to the master network to obtain a different WNNet. This second experiment resulted in an increased presence of master node 3, i.e. its weight was found to be 3, while the other master nodes weights were found to be equal to 1. To construct the corresponding WNNet, two new copies of master node 3, and the necessary edges, were added to the master network (Fig. 2c). Then, degree was computed for each network, i.e. master and two WNNets.

Degree may provide novel, interesting interpretations when used to investigate WNNets. First, the master network was analysed, and master node 3 was found to be the one with the highest number of neighbours (Fig. 2d, left barplot). Then, degree was computed for the first WNNet. Here, weighting master node 5 led to an increased number of neighbours for both master nodes 3 and 4 (Fig. 2d, central barplot). Finally, computing the degree of the second WNNet showed how the increased weight of master node 3 led to a higher number of neighbours for master nodes 2, 4 and 5 (Fig. 2d, right barplot).

## Results

### The idea behind node weighting

The logic behind WNNets was grounded on considerations close to biology. Cell physiology is controlled by biochemical reactions such as signal transduction pathways^34^. These molecular mechanisms rely on an intricate, nonlinear ensemble of binary relationships. Altogether, these interactions generate a network whose architecture can be abstracted as a graph, characterised by very specific properties^35^. Since PPI are mediated by protein domains and motifs, which are both genetically encoded, the properties of a PPI network are genetically determined as well^36,37^. In other words, the characteristics of a PPI network only depend on the proteins that are included in the model, since the set of their interactions is already determined. Thus, independently from the kinetics of a biological event, a set of proteins will be modelled as a network that possesses invariant properties, i.e. the same number of nodes and edges. However, the same set of proteins may eventually show, in different functional contexts, distinct kinetics properties that depend, for instance, on the expression levels of every single protein.

These variables may greatly affect the functional output of PPI networks and should be considered when networks are constructed, and their properties calculated. Indeed, if a cell state, e.g. homeostasis, is controlled by a specific PPI network, and if the proteins involved in the PPI network are quantitatively different in two distinct cellular contexts, i.e. they are highly expressed in cell type A with respect to cell type B, then the corresponding PPI network should be much more functionally active in cell type A than in cell type B. However, current PPI network models do not account for the differential activation state of the proteins in the two cell types. As a consequence, a traditional network analysis does not consider the experimental information that determines the difference between cell A and B. Hence, a linear node weighting procedure, proportional to the experimental data, that incorporates the biological variability into the network structure, as WNNets do, should increase the analytic power of the model.

**Figure 3:**
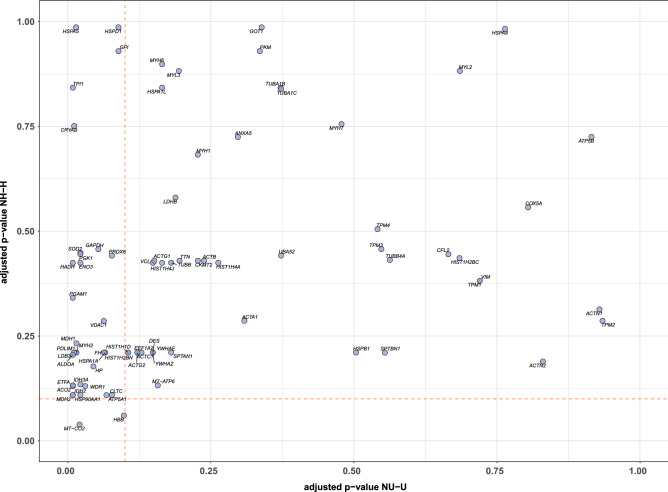
Degrees analysis. The bubble plot shows the adjusted *p-values* that were computed to obtain SSPs for both comparisons, i.e. NH-H and NU-U. All the 79 proteins in the master network are reported. The pink, dashed lines represent the adjusted *p-value* threshold with $FDR < 0.1$. Notably, 2 proteins fell below the horizontal, pink dashed threshold, i.e. H-SSPs for NH-H comparison, while 32 proteins fell below the vertical, pink dashed threshold, i.e. U-SSPs for NU-U comparison. The proteins in the bottom left corner were found to be significant for both comparisons.

### Degree unveils treatment-specific proteins

A semi-quantitative, proteomics data set^18,19^ was used to construct WNNets in a real experimental context. The data set included 12 healthy control samples of myocardial tissue (6 NH samples and 6 NU samples), 6 samples of tissue that suffered a heart attack and were treated with PBS (U samples), and 6 samples of tissue that suffered a heart attack and were treated with FMhMSCs (H samples). Globally, 24 WNNets were constructed, one for each sample, starting from a master network comprising 79 proteins.

Degree was computed for each WNNet to retrieve proteins with a high number of neighbours for the healthy control samples, i.e. NH and NU-ControlPs, and for both the H and U treated samples, i.e. H and U-TreatedPs (Fig. 1). Also, degree was used to test whether a difference existed between healthy controls and treated samples.

Results showed that a treatment-dependent effect existed in WNNets and several SSPs were found. Specifically, 32 U-SSPs (ACO2, SOD2, IDH3A, MYH2, PDLIM1, LDB3, ALDOA, GAPDH, PGK1, ATP5A1, VDAC1, MDH2, HSP90AA1, HSPA1A, ETFA, WDR1, CLTC, MT-CO2, CRYAB, ENO3, PGAM1, HADH, FH, HSPD1, TPI1, MDH1, GPI, PRDX6, HBB, HP, HSPA5, and IDH2) were found for the NU-U samples comparison, while only 2 H-SSPs (MT-CO2 and HBB) were found for the NH-H samples comparison. The two H-SSPs were also found in the U-SSPs set (Fig. 3).

**Figure 4:**
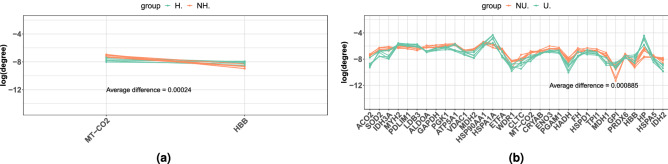
Average degree comparison for SSPs. The line plots show the trends for the average degree computed for the SSPs, whose names are provided on the x axis. Lines representing healthy control samples (NH and NU) are coloured in orange, while lines representing treated samples (H and U) are coloured in green. Average differences are also reported.

Then, the degree of SSPs was analysed for the two comparisons, i.e. NH-H and NU-U (Fig. 4). Results showed that the average degree for the NH-H samples comparison had a very similar trend. In contrast, the degree for NU-U samples comparison showed a greater range of variation. Indeed, treated samples in the NU-U line plot (Fig. 4b) were more dispersed when compared to treated samples in the NH-H line plot (Fig. 4a). Finally, the average differences for the NU-U and NH-H comparisons were compared as $Difference\ Ratio = \frac{ diff _{NU-U}}{ diff _{NH-H}}=\frac{0.000885}{0.00024}=3.6875$.

The results from the random weighted WNNets analysis showed that no SSPs were found, for all the trials. Specifically, for the NU-U random samples comparison, the adjusted *p-values* were almost always higher than 0.95, while, for the NH-H comparison, the adjusted *p-values* had a greater range of variation (see Supplementary materials, Appendix D).

### Degree, experimental significance, and fold change were not always related

To better understand the properties of degree in WNNets, fold change was computed on the set of 79 proteins. Results showed that only 1 protein scored as H-SFCPs for the NH-H samples comparison, while 19 proteins scored as U-SFCPs for the NU-U samples comparison.

**Figure 5:**
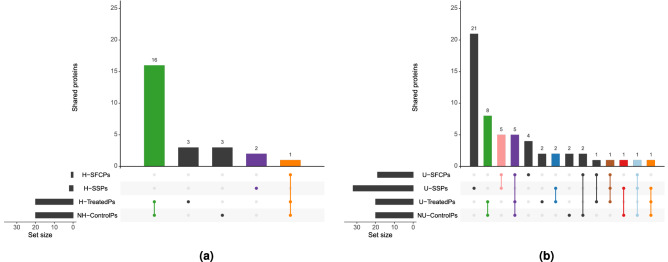
High degree was not always predictive of fold change or significance. Each bar, in each upset plot, represents the number of proteins that belonged to a set or that were shared across two, or more, sets. Sets are listed on the left panel. Set size represents the number of proteins for each of the listed sets. In **(a)**, the comparison between highest scoring proteins in terms of degree (H-TreatedPs and NH-ControlPs), H-SFCPs, and H-SSPs for the NH-H comparison, is shown. In **(b)**, the comparison between highest scoring proteins in terms of degree (U-TreatedPs and NU-ControlPs), U-SFCPs, and U-SSPs for the NU-U comparison, is shown.

Concerning the NH-H samples comparison, the two sets of high-degree proteins, i.e. NH-ControlPs and H-TreatedPs, had 17 proteins in common (Fig. 5a, green and orange bars) of which 1 was shared between these two sets and H-SFCPs (Fig. 5a, orange bar). On the other side, 2 H-SSPs were found and none of them was shared (Fig. 5a, purple bar).

Concerning the NU-U treated samples comparison, 14 proteins were shared between the two sets of high-degree proteins, i.e. NU-ControlPs and U-TreatedPs (Fig. 5b, green, purple, and orange bars). 5 of these proteins were also shared with the set of U-SFCPs (Fig. 5b, purple bar). Also, 7 out of 32 U-SSPs were shared with U-SFCPs (Fig. 5b, pink, brown, and light-blue bars). Finally, 4 and 3 out of 32 U-SSPs were found shared with U-TreatedPs and NU-ControlPs, respectively (Fig. 5b, blue, brown, and orange for U-TreatedPs and red, light-blue and orange bars for NU-ControlPs).

Notably, 14 proteins were shared between all the high-degree proteins sets (see Supplementary materials, Appendix C).

## Discussion

Network analysis is a fast growing area of investigation, and many research groups are actively designing and developing novel methods that can withstand with the ever-increasing complexity of data. The downside of constructing networks with weighted nodes and edges refers to the amount of mathematics and statistics that needs to be considered^13–17^. Indeed, these efforts may result in difficult application and interpretation, making such methodologies complex for non-experts.

In contrast, constructing and investigating the properties of WNNets did not require the adoption of novel techniques, nor the definition of ad-hoc centrality indexes or complex statistical tests. As results have demonstrated, degree allowed to rank proteins and to characterise healthy controls and treated samples. Also, differences were found by comparing the degree between healthy control samples (NH or NU), and the corresponding treated samples (H or U) in terms of SSPs (Fig. 3). The similarities that were found for the NH-H treated samples comparison and the differences between NU-U samples comparison were very interesting (Fig. 4) and might be dependent on the treatments. On the one side, no improvements were expected for the U samples. On the other side, H samples were expected to recover, at least partially, the properties of a healthy tissue, as other works on this data set previously reported^18,19^. Notably, the construction of the master network was a trivial step. Indeed, two approaches were available. The first consisted in removing those proteins with SpC equal to zero, group by group, i.e. NH, NU, U and N or sample by sample. The other consisted in removing all the proteins with a single SpC equal to zero across the whole data set. The first option would lead to different masters networks, one for each experimental group or each sample. The second option would lead to a single master network comprising only those proteins that were shared across all the 24 samples. Clearly, using a single, shared master network allowed one to focus on the consequences of master nodes weighting only, while analysing different masters networks meant that the properties of the WNNets would also be affected by the different number of master nodes and edges in the master networks. In other words, the differences that were found using WNNets (Figs. 3,  4) constructed from a common master network were direct consequence of the weighting and did not depend on different master networks structures.

Results also showed that high-degree proteins, i.e. TreatedPs and ControlPs, were not always the proteins of choice to distinguish between healthy controls and treated samples. Indeed, 0 H-SSPs and 6 out of 32 U-SSPs were shared with the corresponding set of high-degree proteins, i.e. TreatedPs or ControlPs. In contrast, 17 out of 20 H-TreatedPs, and 14 out 20 U-TreatedPs were shared with the corresponding set of ControlPs, i.e. NH or NU (Fig. 5). Also, most of the high-degree proteins that were considered, were found shared between all healthy controls and treated samples (see Supplementary materials, Appendix C). These results suggested that the global structure of the network was not affected by master nodes weighting, in terms of high degree master nodes, i.e. hubs. Eventually, a hub remained a hub. In contrast, the fact that only a fraction of SSPs was shared with its corresponding set of high-degree proteins, i.e. ControlPs and TreatedPs, (Fig. 5) suggested that the degree of a master node was not a good metric to measure the relevance of a protein. Indeed, as results shown, weighting master nodes had very interesting effects on the local neighbourhoods. As showed, in WNNets, the degree of a master node did not depend on the experimental value of the master node itself. Instead, it was determined by the experimental values of its neighbours (Fig. 2). This was a fundamental finding since, in principle, the more a protein is expressed, the more it should influence its neighbours and, as a consequence, its regulatory importance increases. However, WNNets highlighted the opposite behaviour, i.e. the relevance of a master node in a WNNet was influenced by the experimental values of its neighbours and not by its own weight.

**Figure 6:**
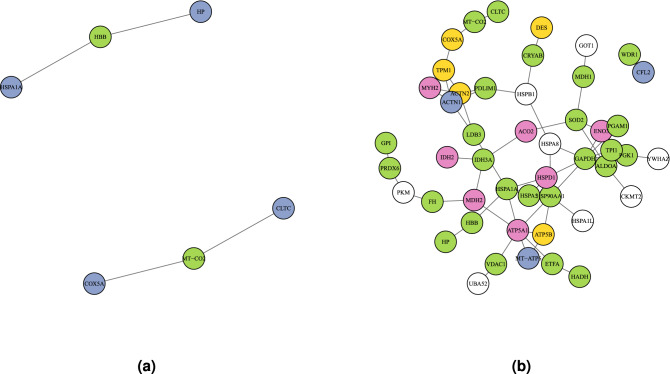
SSPs subnetworks. These networks represent the interactions between SSPs and their first neighbours, for both H **(a)** and U **(b)** groups, respectively. SSPs are coloured in green, SFCPs are coloured in yellow, and proteins belonging to both sets are coloured in pink. Proteins that contributed to SSPs or SFCPs degree are coloured in purple.

In addition, the fact that a few SSPs were found shared with their corresponding set of SFCPs suggested that treatment-specific proteins, i.e. SSPs, were not detected using fold change. This fact suggests that a protein with an increased number of copies, i.e. an SFCP, may influence its surroundings and potentially trigger the emergence of treatment-specific proteins, i.e. SSPs. Interestingly, results showed that SSPs and SFCPs tend to be neighbours (Fig. 6). Moreover, the analysis revealed the presence of few master nodes, tightly connected to SSPs, that played an important role in determining the relevance of their neighbours (Fig. 6, in purple). Indeed, these master nodes, that were directly interacting with an SSP, might be promising targets since they actively contributed in defining the SSPs degree.

The data that were used to show how WNNets were constructed and analysed, were semi-quantitative proteomics data and, even though SpC is not a perfect measure for quantifying proteins, it is widely used in proteomics studies^38^. Also, the same data were used for two different works^18,19^ and WNNets results were coherent with what was previously reported. Clearly, using quantitative information may surely enhance the predictive power of WNNets but it is important to remark that the methodology is very flexible and that master nodes may be weighted using many different measures depending on the field of application such as, for instance, gene expression levels.

Finally, to test the reliability of WNNets, a comparison against randomly generated weighting data sets was performed. As results showed, out of 100 random experiments, no protein was found to be SSP (see Supplementary materials, Appendix D). These results were a further indication that the numerical values, i.e. the weights, that were used to multiply the master nodes in each WNNet contained valuable information since, using randomised data set, adjusted *p-values* were always negligible. In other words, investigating real data by means of WNNets may provide biologically relevant insights that strongly depend on the numerical, experimental information used to weigh the master nodes. It is important to note, however, that an edge weighting methodology based on, for example, gene expression data, may allow for a finer biological tuning of a WNNet and, possibly, lead to even more significant results. Finally, novel opportunities may emerge when it will be possible to include, in this analysis, other established centralities such as stress or centroid.

In conclusion, a novel network model was designed and developed. It has direct application to the field of PPI networks and, more in general, of omics modelling. WNNets may be adopted in many other different contexts such as time series of networks where node status changes over time and where networks should be compared against each other. In the present work, treatment-specific, weighted nodes were easily retrieved using a well-known centrality index, i.e. degree. Strong mathematical foundations for WNNets were built, guaranteeing that network centralities were not affected by node addition (see Supplementary materials, Appendix A). Finally, a software that enables the construction and analysis of WNNets is missing, but a novel implementation of CentiScaPe^39,40^ incorporating this new methodology is under development.

## Supplementary Information

Supplementary Information 1.

Supplementary Information 2.

## Data Availability

Appendix A and Appendix B contain further mathematical details about WNNets. Some supplementary figures are also provided. The data set and code are available at https://bitbucket.org/gabrielet/wnnets/src/master/.
