# Supplementary material for: Analysing omics data sets with weighted nodes networks (WNNets)
Source: Sci Rep. 2021 Jul 14;11:14447. doi: 10.1038/s41598-021-93699-3 (PMC8280138; doi:10.1038/s41598-021-93699-3)
Supplement: Supplementary file 2 — Supplementary Information 2. [file 41598_2021_93699_MOESM2_ESM.pdf]

Analysing omics data sets with weighted nodes networks (WNNets)

Gabriele Tosadori[1,2*], Dario Di Silvestre[4], Fausto Spoto[3], Pierluigi Mauri[4], Carlo Laudanna[2*+], and Giovanni Scardoni[1+]

[1]University of Verona, Center for BioMedical Computing (CBMC), Strada le Grazie 8, 37134, Verona, Italy
[2]University of Verona, Department of Medicine, Section of General Pathology, 37134, Verona, Italy
[3]University of Verona, Department of Computer Science, Strada le Grazie 15, 37134, Verona, Italy
[4]Institute for Biomedical Technologies, National Research Council (ITB-CNR),via F.lli Cervi 93, 20090, Segrate, Milan, Italy
[*]gabriele.tosadori@gmail.com, carlo.laudanna@univr.it
[+]Co-last author

ABSTRACT

Current trends in biomedical research indicate data integration as a fundamental step towards precision medicine. In this context, network models allow representing and analysing complex biological processes. However, although effective in unveiling network properties, these models fail in considering the individual, biochemical variations occurring at molecular level. As a consequence, the analysis of these models partially loses its predictive power. To overcome these limitations, Weighted Nodes Networks (WNNets) were developed. WNNets allow to easily and effectively weight nodes using experimental information from multiple conditions. In this study, the characteristics of WNNets were described and a proteomics data set was modelled and analysed. Results suggested that degree, an established centrality index, may offer a novel perspective about the functional role of nodes in WNNets. Indeed, degree allowed retrieving significant differences between experimental conditions, highlighting relevant proteins, and provided a novel interpretation for degree itself, opening new perspectives in experimental data modelling and analysis. Overall, WNNets may be used to model any high-throughput experimental data set requiring weighted nodes. Finally, improving the power of the analysis by using centralities such as betweenness may provide further biological insights and unveil novel, interesting characteristics of WNNets.

1 Supplementary materials

1.1 Appendix A - Weighting by preserving shortest paths

In a PPI network, shortest paths represent flows of information between nodes and are used to establish which nodes are more important for the network functioning[1]. Centrality indexes like stress, betweenness, and bridging count the number of shortest paths passing through a node, while centralities like closeness and diameter measure the length of the shortest paths to infer both nodes and network characteristics. Hence, assuring that the information flow, in terms of shortest paths, was not affected by the addition of new nodes and edges was of fundamental importance. Here, a mathematical proof is provided demonstrating that the addition of k copies of node v to the master network G did not generate new shortest paths in the resulting WNNet G', that were shorter or longer than any of the shortest paths already existing in G.

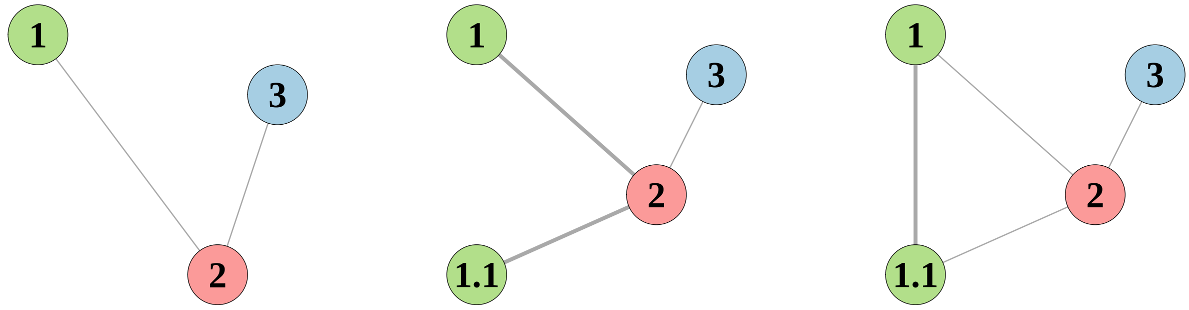

(a) (b) (c)

Figure 1. Why edges connecting the copies are needed and how degree works. A minimal, master network, with three nodes (Figure 1a), is weighted in the wrong way (Figure 1b). Indeed, a copy of the node 1, i.e. node 1.1 in green, was included in the master network but no edge connecting node 1 and 1.1 was added. So, a new shortest path, i.e. the thick edges, connecting node 1 and 1.1, emerged. This new path, i.e. node 1 - node 2 - node 1.1, was not present in the master network (Figure 1a) since node 2 only connects node 1 with node 3, hence its structure is now lost. Such inconsistency was prevented by adding an edge connecting the master node 1 and 1.1 (Figure 1c). Here, the thick edge connected node 1 and 1.1 and no new shortest path, passing through a third node, emerged.

To achieve this goal a graph $G = (V,E)$ and its corresponding WNNet, defined as $G' = (V',E')$, were constructed (for further details about WNNets construction, see the main text, *Methods* section, *Definition of WNNets* subsection). In addition, a shortest path $p = (u,...,x,v_i,y,...,z) \in G \mid (u,x,v_i,y,z) \in V$ was defined.

At this point, a copy z' was added to G and a shortest path $p' \in G'$ connecting $u,z' \in V'$ was generated, such that $length(p')$ is lower than the length of any path between u and z in G. Necessarily, p' passed through one of the nodes v_c in $Clique = \{v'_1,\dots,v'_a,v_i\}$ and two of its incident edges, such that $p' = (u,...,x,v_c,y,...z')$, for some $x,y \in V$. Note that the edges (x,v_c) and (v_c,y) existed in G since $(x,v_c) \in E'$ and $(v_c,y) \in E'$. Clearly, $length(p) = length(p')$ hence the assumption, i.e. that a new shortest path, shorter or longer, has been generated by adding a new node, was wrong.

To further clarify this demonstration, a minimal example was provided. The starting point was the master network with three master nodes and two edges connecting them (Figure 1a). Then, a copy of the master node 1, i.e. copy 1.1, was added and connected with master node 2, i.e. the only neighbour of master node 1. As a consequence, a new shortest path connecting master node 1 with its copy, i.e. copy 1.1, was created (Figure 1b). But, such a shortest path did not belong to the master network. Indeed, in the master network, master node 2 connected master node 1 with master node 3. Hence, the existing shortest paths were (1, 2), (2, 3) and (1, 2, 3). When copy 1.1 was added without connecting it to its master node (Figure 1b) a new shortest path, i.e. (1, 2, 1.1), was created. In contrast, connecting master node 1 with its copy did not create any new shortest path connecting each other. In other words, copy 1.1 is now directly interacting its master node and its neighbours, i.e. master node 2, without generating new, unexpected, shortest paths. However, a new shortest path was generated, i.e. (1.1, 2, 3), but this is a copy of a shortest path which was already present in the master network hence the type of shortest paths and their length were preserved.

1.2 Appendix B - Weighting by using any positive number in $\mathbb{R}$

In the manuscript, the theoretical framework of WNNets was described and mathematical proof of the weighting methodology, in terms of shortest paths, was provided. Once it was guaranteed that shortest paths were not affected by node weighting, it was possible to expand the WNNets theoretical framework to include weights belonging to the set of positive real numbers, $w_p \in R_{>0}, \{n \in \mathbb{R} \mid n > 0\}$. As discussed in the main text *Methods* section, *Definition of WNNets* subsection, the main assumption of WNNets concerned the fact that each node weight $w_p = 1 \mid v_p \in V'$. However, in real world data sets, this is not always the case and, as an example, few ad-hoc experiments were designed to show how degree works even with positive real numbers (Figure 2). Indeed, the degree of a node, in WNNets, only represents the sum of experimental quantities, i.e. the master nodes weights or protein abundances, of the neighbours a master node has. From a mathematical perspective, it is not important whether these quantities are natural, rational or real numbers.

In these ad-hoc experiments, three proteins were detected, and their quantities represented by positive real numbers. In the first experiment, the amount of each protein was found equal to 1 (Figure 2a). In the second experiment, the abundance of P1 was found equal to 4, P2 was found equal to 1, and P3 was found equal to 3 (Figure 2b). Finally, in the third experiment, the abundance of P1 was found equal to 4.5, P2 was found equal to 1.5, and P3 was found equal to 3.5 (Figure 2c). This last experiment does not fit in the general, theoretical framework that assumes weights to be always natural numbers. But, the degree computation was not affected and the pink master node increased its importance thanks to the contribution of each one of its neighbours (Figure 2d).

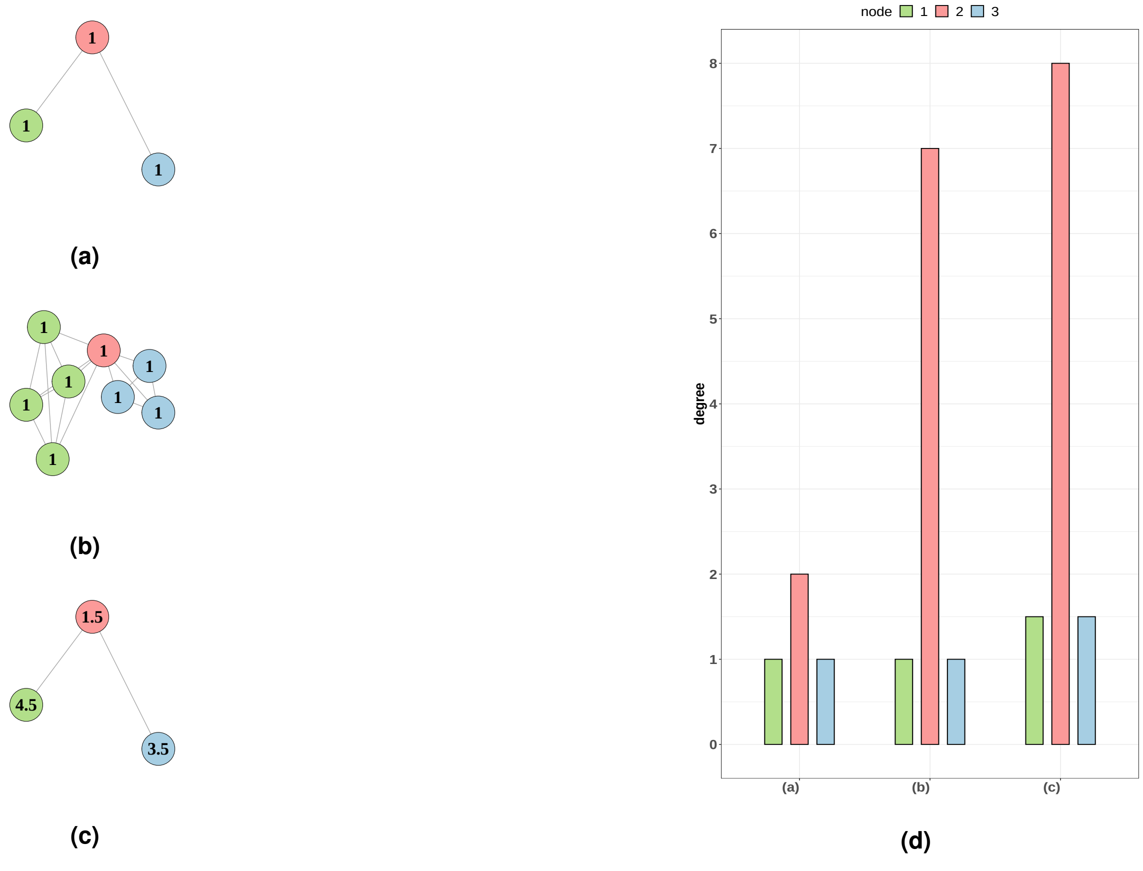

Figure 2. Weighting with decimals. Three ad-hoc experiments are shown. Node labels represent nodes weights, for three proteins, i.e. P1 in light-green, P2 in pink, and P3 in light-blue. The master network (Figure 2a) was multiplied using natural numbers (Figure 2b) and positive real numbers (Figure 2c). For each network, degree was computed and represented in the bar plots (Figure 2d). Notably, it was possible to represent P1, P2, and P3 through master nodes and copies for the first and second experiment only (Figure 2a and Figure 2b). In contrast, positive real weights were used as labels for P1, P2, and P3, in the third experiment, since it was not possible to represent a fraction of a node, e.g. P3 through 3.5 nodes, by using a master node and some copies. However, the representation has no influence on how the degree is eventually computed.

1.3 Appendix C - High degree, SSPs and SFCPs

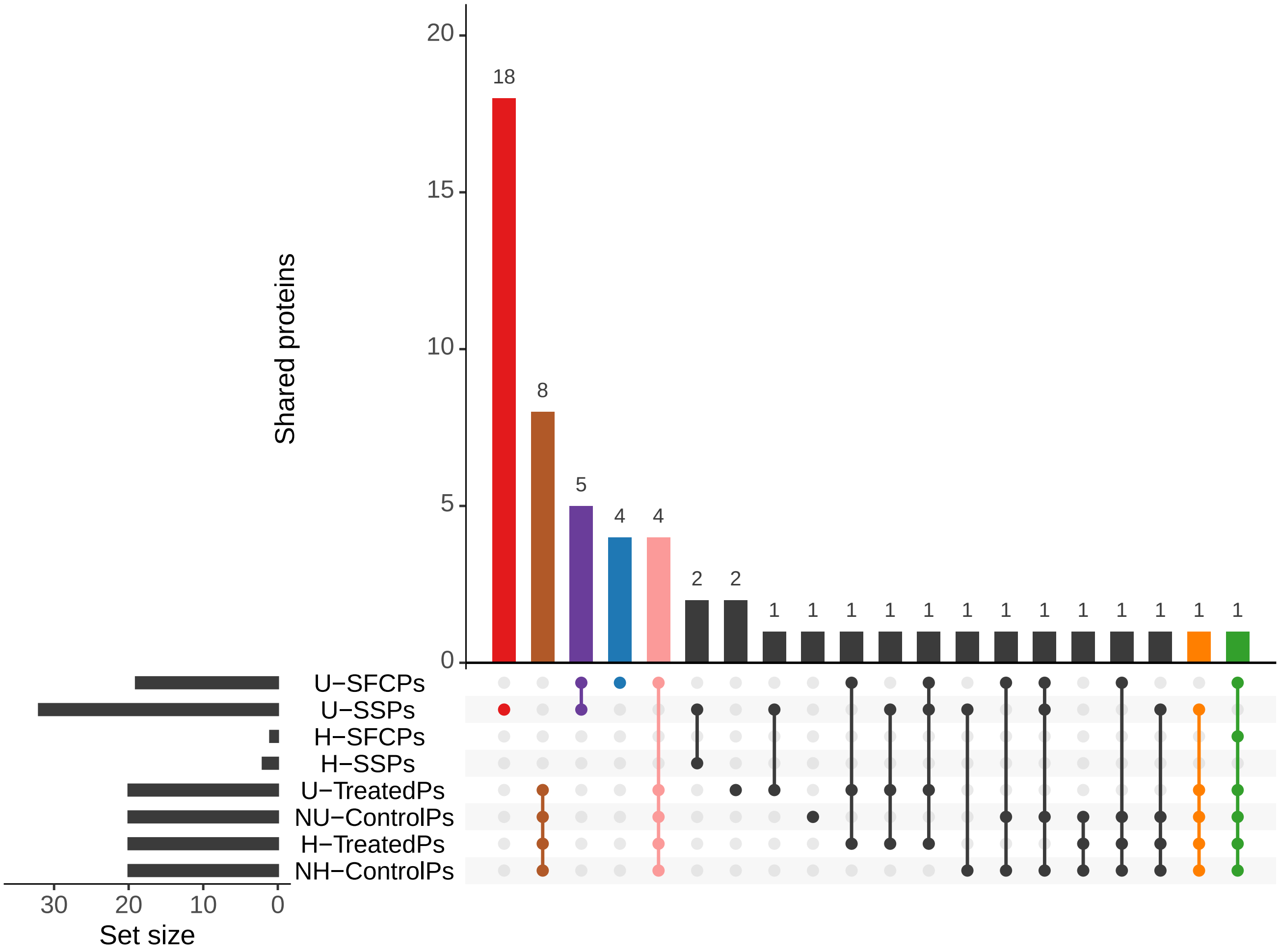

Figure 3. Full comparison between all the sets. The upset plot shows the comparison between all the different sets of proteins that were obtained using WNNets and proteomics data, i.e. high degree proteins (ControlPs and TreatedPs), statistically significant proteins (SSPs), and high fold change proteins (HFCPs).

1.4 Appendix D - Finding SSPs by comparing randomised WNNets

To test the reliability of WNNets, a comparison using randomly generated weighting data sets was performed. To achieve this goal, 100 random weighting data sets were generated, comprising 79 rows, one for each protein, and 24 columns, one for each sample. Then, each of these 100 data sets was used to construct a set of WNNets by multiplying the master nodes in the original master network. Eventually, 24 randomly weighted WNNets were built and used to perform the two comparison, i.e. NH-H and NU-U, as described in the manuscript. Then, SSPs were retrieved applying multiple tests correction, using False Discovery Rate (FDR). The same procedure was applied for each trial.

Results showed that the differences between WNNets, in terms of SSPs, were almost always negligible (Figure 4a and Figure 4b), i.e. no SSPs were found. Interestingly, adjusted *p-values* for the NH-H comparison had a greater range of variation and only two proteins (LDHB and HBB) reached the significance threshold, i.e. $FDR < 0.1$, in one trial (Figure 4a, left plot).

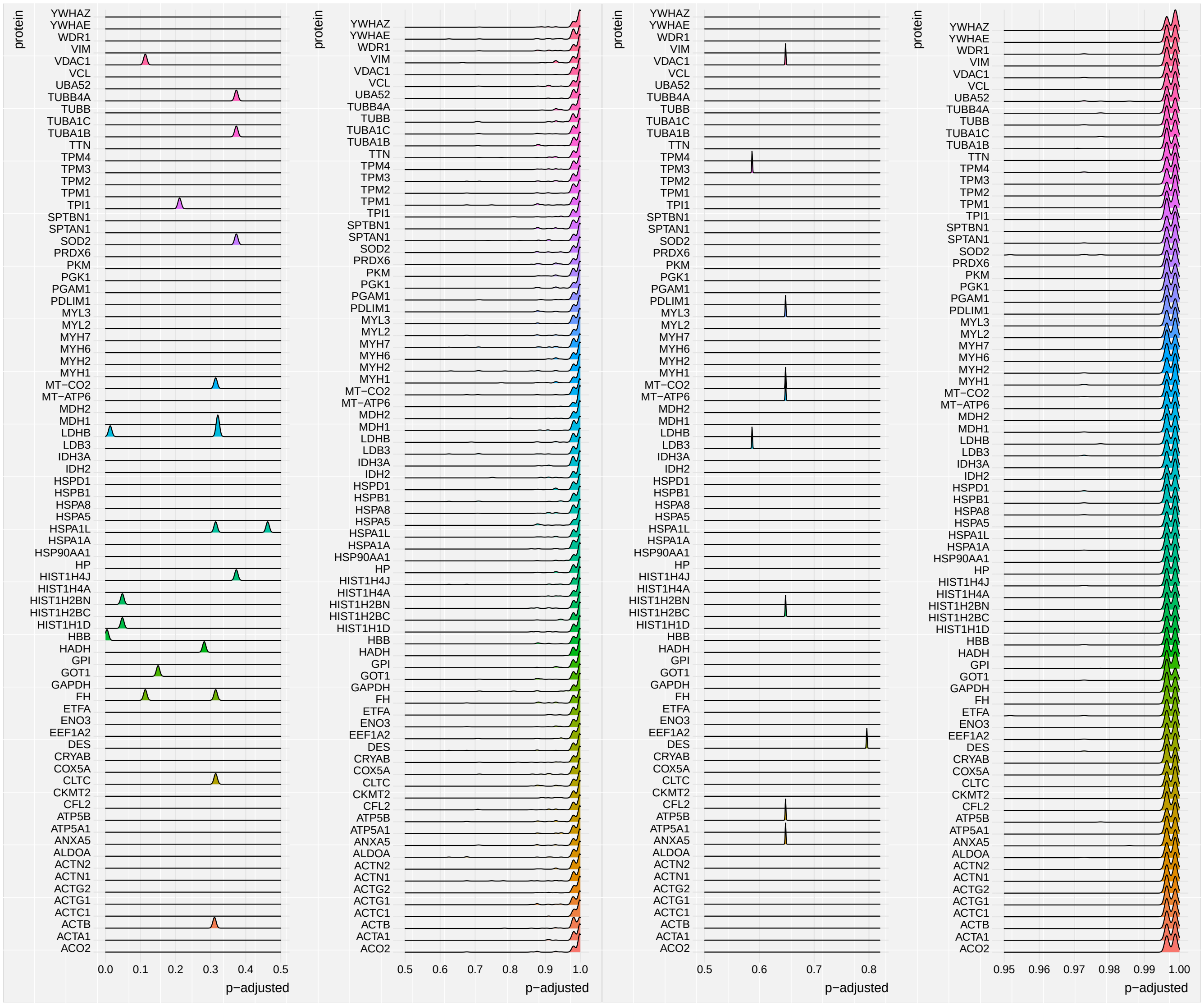

(a) **(b)**

Figure 4. **SSPs frequency for adjusted *p-values*.** The ridge plots show the FDR-adjusted *p-values* obtained by comparing the differences, in terms of average degree, across 100 random trials. For the comparison between NH-H (Figure 4a), two ridge plots were reported to show all the adjusted *p-values*. The same two ridge plots were reported for the NU-U comparison (Figure 4b). The x-axis range of each plot is different to allow for a better representation of the data.

1.5 Appendix E - Master network

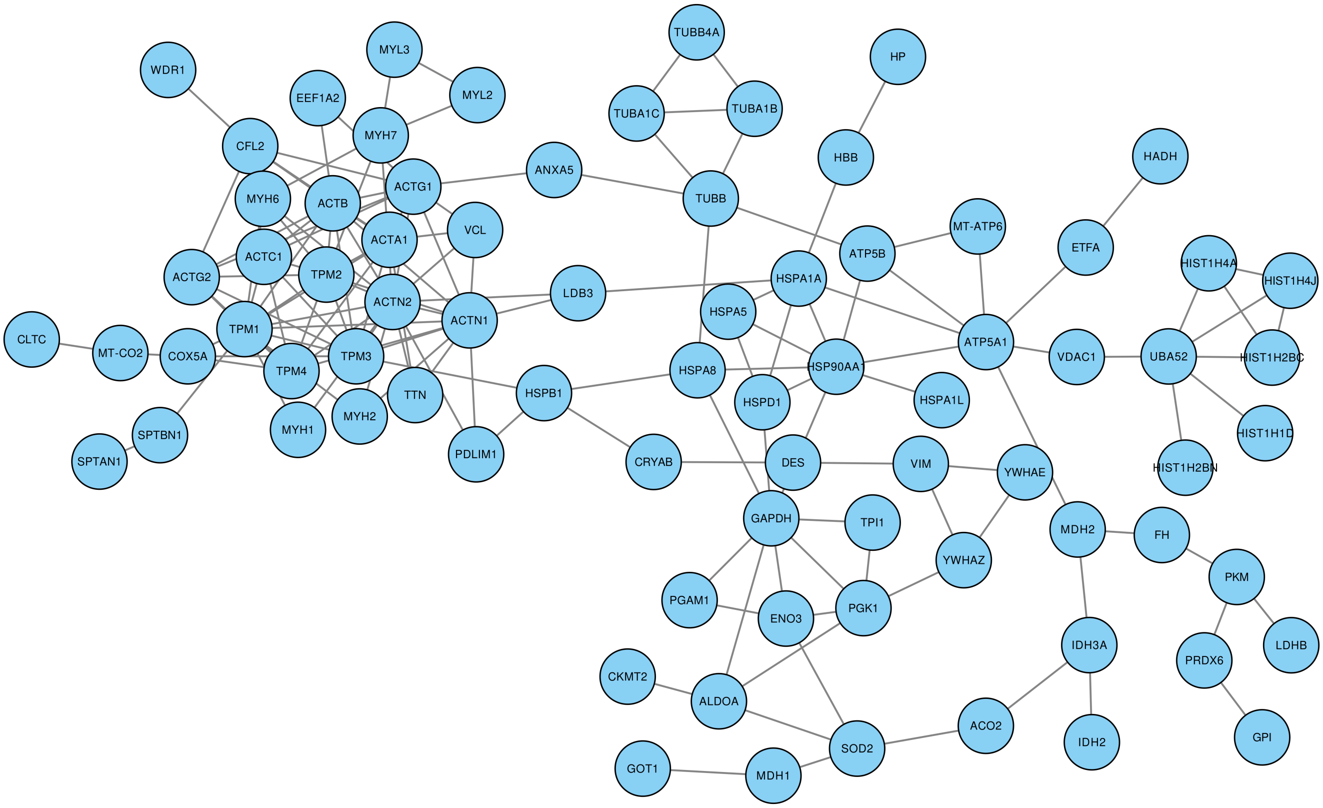

Figure 5. Master network. All the 79 nodes and the 155 edges in the master network.

References

1. Pavlopoulos, G. A. *et al.* Using graph theory to analyze biological networks. *BioData mining* **4**, 1–27 (2011).
